# The effect of age on the clinical presentation and treatment of women with psychosis: secondary analysis of the IMPaCT Randomised Clinical Trial

**DOI:** 10.1192/bjo.2025.10860

**Published:** 2025-12-02

**Authors:** Maria Antonietta Nettis, Khalida Ismail, Robin M. Murray, Aikaterini Dima, Kathryn Greenwood, Zerrin Atakan, Shubulade Smith, Fiona Gaughran

**Affiliations:** South London and Maudsley NHS Foundation Trust, London, UK; Department of Psychosis Studies, Institute of Psychiatry, Psychology and Neuroscience, King’s College London, London, UK; Department of Psychological Medicine, Institute of Psychiatry, Psychology and Neurosciences, King’s College London, London, UK; Institute of Psychiatry, Psychology and Neuroscience, King’s College London, London, UK; Oxleas National Health Service Foundation Trust, London, UK; Sussex Partnership NHS Foundation Trust and School of Psychology, University of Sussex, Brighton, UK; Department of Forensic and Neurodevelopmental Science, Institute of Psychiatry, Psychology and Neuroscience, King’s College London, London, UK; National Institute for Health Research, Maudsley Biomedical Research Centre, South London and Maudsley National Health Service Foundation Trust, London, UK; National Institute for Health Research Applied Research Collaboration at King’s College Hospital NHS Foundation Trust, London, UK

**Keywords:** Women, psychosis, age, menopause, prolactin

## Abstract

**Background:**

According to existing evidence, during menopause transition, women with psychosis may present with exacerbated psychiatric symptoms, due to age-related hormonal changes.

**Aims:**

We aimed to (a) replicate this evidence, using age as a proxy for peri/menopausal status; (b) investigate how clinical presentation is affected by concomitant factors, including hyperprolactinaemia, dose and metabolism of prescribed antipsychotics using cross-sectional and longitudinal analyses.

**Method:**

Secondary analysis on 174 women aged 18–65, from the IMPaCT (Improving physical health and reducing substance use in psychosis) randomised controlled trial. We compared women aged below (*N* = 65) and above 40 (*N* = 109) for (a) mental health status with the Positive and Negative Syndrome Scale (PANSS) and Montgomery Asberg Depression Rating Scale; (b) current medications and (c) prolactin levels, at baseline and at follow-up (12/15 months later).

**Results:**

Women aged above 40 showed higher baseline PANSS total score (mean ± s.d. = 53.4 ± 14.1 *v*. 48.0 ± 13.0, *p* = 0.01) and general symptoms scores (28.0 ± 7.4 *v*. 25.7 ± 7.8, *p* = 0.03) than their younger counterparts. Progressive sub-analysis revealed that this age-related difference was observed only in women with non-affective psychosis (*n* = 93) (PANSS total score: 57.1 ± 13.6 *v*. 47.0 ± 14.4, *p* < 0.005) and in those prescribed antipsychotic monotherapy with olanzapine or clozapine (*n* = 25) (PANSS total score: 63 ± 16.4 *v*. 42.8 ± 10.9, *p* < 0.05).

Among all women with hyperprolactinaemia, those aged above 40 also had higher PANSS positive scores than their younger counterparts. No longitudinal differences were found between age groups.

**Conclusions:**

Women aged above 40 showed worse psychotic symptoms than younger women. This difference seems diagnosis-specific and may be influenced by antipsychotics metabolism. Further longitudinal data are needed considering the menopause transition.

In women with psychotic illnesses, psychiatric presentation and response to medications can be influenced by age and associated hormonal status.^1^ This is because, aside from evolving family and social demands, oestrogen levels change throughout life, affecting both mental health and metabolic pathways.

## Oestrogen, menopause transition and antipsychotics

Oestrogen not only has neuroprotective effects, but also the ability to increase D2 receptor sensitivity and occupancy by antipsychotic medications.^2,3^ Moreover, as previously suggested,^4^ oestrogen levels have differing effects on the pharmacokinetics of some antipsychotics. In women of reproductive age, oestrogens can accelerate, to a various extent, the metabolism of antipsychotics such as risperidone, quetiapine, haloperidol and lurasidone by inducing the liver enzyme cytochrome P-450 (CYP) 3A4. By contrast, oestrogen inhibits the enzyme CYP 1A2, which metabolises olanzapine and clozapine, resulting in higher blood levels of these drugs.

These factors become especially relevant during the menopause transition, when oestrogen levels decrease significantly. Consequently, the oestrogen neuroprotective effect is reduced, with a resulting alteration in the antipsychotic metabolism and thus drug concentration and, potentially, effectiveness or tolerance. Therefore, perimenopausal and menopausal women may be at higher risk of psychotic relapse or the adverse effects of antipsychotic medication. Indeed, women with psychotic illnesses in the perimenopausal and menopausal period exhibit worse psychiatric symptoms,^5^ higher hospitalisation rates^4^ and reduced effectiveness of antipsychotic medications compared to men and premenopausal women.^6^ However, to date, quantitative research on menopause in women with serious mental illness is limited, as is the availability of longitudinal data on symptoms course and medication response.

## The role of prolactin

The clinical picture is further complicated as medicated women with psychosis may experience a hypoestrogenic state with oligo- or amenorrhoea due to antipsychotic-related hyperprolactinaemia. This can be associated with ovulatory dysfunction and gonadal suppression. Whether hyperprolactinaemia contributes to exacerbation of psychosis, and whether its effect varies with age and menopause status, is not clear, as research on the topic is scarce and results conflicting.^3,7^ However, in a recent longitudinal study in first-episode (non-affective) psychosis (FEP), Jordà-Baleri et al demonstrated sex-specific associations: in males, prolactin mediated the relationship between negative symptoms and functional outcome; in females, prolactin negatively correlated with cognitive performance.^8^

## Aims and hypotheses

Considering the above, this study aimed to analyse data from an existing dataset to explore the relationship between age, prolactin levels, prescribed antipsychotics and psychiatric presentation in a cohort of women with psychosis. We hypothesised that in our sample of women, older age or presenting with hyperprolactinaemia (at any age) would be associated with more severe psychotic and affective symptoms and worse illness course.

## Method

This study is a secondary analysis of data from the IMPaCT randomised controlled trial (RCT) (Improving physical health and reducing substance use in psychosis). IMPaCT was a multicentre, two parallel-arm, cluster design RCT,^9^ integrated within UK community mental health teams (CMHTs). It focused on the effect of training and supervising staff to work with their patients with psychosis, using a motivational interview-based Health Promotion Intervention. Data were collected at baseline (0) and at 12- and 15-months follow-up on both physical and mental health outcome measures.

### Participants

Details on recruitment are fully described elsewhere,^10^ but we summarise them here. The multi-site RCT recruited care co-ordinators across (non-early intervention) CMHTs in five National Health Service Mental Health Trusts, who were working with eligible patients. Participants were eligible if they were aged between 18 and 65 years with an established diagnosis of a psychotic disorder (ICD-10 diagnosis F20–29, F31.2, F31.5, F32.3 or F33.3)^11^ and had capacity to consent to the study. Exclusion criteria included (a) a primary diagnosis of an intellectual disability, (b) a pre-existing physical health problem that would independently impact on metabolic measures (as judged by medical investigators), (c) current pregnancy or being less than 6 months post-partum or (d) a life threatening or terminal medical condition.

Overall, 454 patients of 104 care co-ordinators consented to be enrolled. Four hundred and six patients were randomised into the trial, with 314 patients completing at least one follow-up timepoint.

For this secondary analysis, we included women participants aged 18 to 60 (we excluded those above 60 for increased risk of dementia, which in people with serious mental illness may appear earlier than in the general population^12^). We also excluded women prescribed hormone replacement therapy (HRT). The final sample included 174 women.

For our analysis, we divided our sample into two groups by age, using the age of 40 as a cut-off. This age is considered a threshold for important biological changes in a woman’s life and for the onset of perimenopausal symptoms.^5,13^ We used this age threshold to account for the 2–8% of women experiencing early menopause (between 40 and 45),^14^ but more importantly, to reflect the diverse ethnicity of our sample. Indeed, women from some ethnic minorities tend to experience the menopause transition earlier than Caucasian women.^15–17^

### Timepoints

We took data at baseline for cross-sectional analysis. For longitudinal analysis, as some participants only had one follow-up point, we included data from either the 12- or 15-month follow-up point, whichever was later and created a ‘combined follow-up’ timepoint.

### Measures

- Sociodemographic data (at baseline): age, sex, self-reported ethnicity.
- Clinical diagnosis according to ICD-10.
- Mental health status (at both timepoints): using the Positive And Negative Syndrome Scale (PANSS) and its three subscales: positive (delusions, conceptual disorganisation, hallucinatory behaviour, excitement, grandiosity, suspiciousness/persecution, hostility), negative (blunted affect, emotional withdrawal, poor rapport, passive apathetic social withdrawal, difficulty in abstract thinking, lack of spontaneity and flow of conversation, stereotyped thinking) and general symptoms (somatic concern, anxiety, guilt feelings, tension, mannerisms and posturing, depression, motor impairment, uncooperativeness, unusual thought content, disorientation, poor attention, lack of judgement and insight, disturbance of volition, poor impulse control, preoccupation or active social avoidance)^18^ and the Montgomery Asberg Depression Rating Scale (MADRS).^19^
- The ‘period problems’ item (at both timepoints) from the self-administered Liverpool University Neuroleptic Side Effect Rating Scale (LUNSERS).^20^ The LUNSERS is designed to assess the perceived side-effects of psychotropic medications across multiple domains. It includes a question on whether the participant has experienced (unspecified) period problems on a 5-point Likert scale: 1 ‘not at all’, 2 ‘very little’, 3 ‘a little’, 4 ‘quite a lot’ and 5 ‘very much’. We reduced the Likert scale to 3 points by combining points 2 and 3 and points 4 and 5, for sample size reasons.
- Anthropometric measurements (body mass index (BMI)) and the inflammatory marker, C-reactive protein.^9^
- Substance use measures: Alcohol use was recorded using the Alcohol Use Disorders Identification Test,^21^ tobacco use with the Nicotine Dependence Questionnaire,^22^ while use of cannabis and other illegal substances (opiates, methamphetamine and cocaine) was recorded using the Time Line Follow Back.^23^

### Prolactin

Prolactin levels at both timepoints (not all participants in the trial consented to the blood test element) was included as both a continuous and a dichotomous variable. We used a cut-off of 1000 mlU/L to indicate moderate hyperprolactinaemia, often associated with oligomenorrhoea.^24^

### Antipsychotic medications

Information on antipsychotic medications^9^ (name, dose, frequency, duration and formulation) was collected at baseline and ‘combined’ follow-up.

#### Prolactin raising versus prolactin sparing

Antipsychotics were divided into prolactin-raising (first-generation antipsychotics, risperidone, paliperidone and amisulpride) and relatively prolactin-sparing (clozapine, quetiapine, olanzapine and aripiprazole) according to the *Maudsley Practice Guidelines for Physical Health Conditions in Psychiatry* ^25^ (Table 1).

**Table 1:** Baseline descriptive demographic, physical and medication variables and associated statistics by age group

Variable	Age ≤ 40 (Total *N* = 65)		Age > 40 (Total *N* = 109)		
	*N*	Data	*N*	Data	Statistics
BMI (median (IQR)	59	32.0 (10)	93	33.0 (12)	*p* > 0.05
C Reactive protein, mg/L (median (IQR))	30	3.7 (6.7)	54	4.4 (8.7)	*p* > 0.05
Olanzapine dose equivalents (median (IQR))	60	16.1 (9.8)	89	15.0 (16.7)	*p* > 0.05
AP(s) treatment duration, days (mean (s.d.))	62	170.8 (32.2)	96	176.5 (24.2)	*p* > 0.05
PRL, mlU/L (median (IQR))	47	288.0 (975)	72	299 (547.75)	*p* > 0.05
PRL >1000 mlU/L (% (*n*))	47	25.5 (12)	72	12.5 (9)	*χ* ^2^ = 3.32 *p* = 0.07
PRL raising APs (% (*n*))	64	39.1 (25)	103	45.6 (47)	*p* > 0.05
Period problems (% (*n*))	60		68		*p* > 0.05
Not at all		68.3 (41)		54.4 (37)	
A little		10 (6)		14.7 (10)	
A lot		21.7 (13)		30.9 (21)	
Prescribed with clozapine or olanzapine (CYP 1A2)* (% (*n*))	52	42.3 (22)	79	29.1 (23)	*p* > 0.05
Current smoker (% (*n*))	65	47.7 (31)	104	52.9 (55)	*p* > 0.05
Duration of illness, years (median (IQR))	57	10.0 (7.50)	82	16.50 (17.25)	***U* = 1515.0** ***z* = −3.50** ***p* < 0.001**
Affective diagnosis** (% (*n*))	63	39.7 (25)	101	40.6 (41)	*p* > 0.05

BMI, body mass index; IQR, interquartile range; APs, antipsychotics; PRL, prolactin levels.

Significant statistics are in bold.

*Women on one antipsychotic only.

**Schizoaffective, bipolar disorder, depressive disorder or other affective diagnosis as opposed to non-affective psychoses.

#### CYP 3A4 versus CYP 1A2

Two further subgroups of antipsychotic prescriptions were created: (a) metabolised partially or totally by CYP 3A4 (risperidone, lurasidone, aripiprazole, haloperidol and quetiapine) and (b) primarily metabolised by CYP 1A2 (clozapine and olanzapine).^26^ To limit the confounding effect of multiple medications, we explored the role of these two groups of antipsychotics only in the subset of individuals on antipsychotic monotherapy (i.e. excluding those on two or more antipsychotics) (see Table 1).

### Statistical analysis

Descriptive differences in categorical variables between the two age groups (below and above 40) were tested with Pearson’s chi square test. Descriptive differences in continuous variables (BMI, olanzapine equivalents, etc.) were tested with non-parametric (Mann–Whitney) tests due to the skewedness of some variables. When we found a between-groups statistically significant difference in descriptive variables, we tested its effect on the outcome variable of our analysis (clinical scores) with a linear regression analysis.

In cross-sectional analysis, we compared baseline PANSS and MADRS scores (dependent variables) between age groups (predictor). We used Mann–Whitney tests, because some clinical variables did not present a normal distribution, despite the absence of extreme outliers.

We performed explorative subgroup analyses comparing four groups, according to age (below and above 40) and diagnostic categories: affective (schizoaffective disorder, bipolar disorder or major depressive disorder with psychotic symptoms) and non-affective psychoses (schizophrenia, delusional disorder, acute psychotic disorder and unspecified non-organic disorder). The resulting four groups were: (a) younger – below 40 – with affective psychosis, (b) younger with non-affective psychosis, (c) older – above 40 – with affective psychosis and (d) older with non-affective psychosis). We employed a Kruskal–Wallis test followed by Bonferroni post hoc analysis to test differences in clinical symptoms among these four groups. We performed the same test in two further subgroups of women on antipsychotic monotherapy with CYP 3A4 versus CYP 1A2.

In longitudinal analysis, we compared, over time, clinical changes in the two age groups with Mann–Whitney tests, while within-group changes were explored with Wilcoxon tests. Exploratory analyses of the relationship with diagnosis and an antipsychotic metabolism pathway were also performed.

We re-ran each analysis with the corresponding parametric test (i.e. *t*-tests, one-way ANOVA and paired *t*-tests) to check the consistency and robustness of results.^27^ Statistical analysis were performed using IBM SPSS Statistics 29.0.1.0 for macOS and Windows (IBM Corporation, Armonk, New York, USA; https://www.ibm.com/products/spss-statistics). Inkscape 1.4.2 for macOS (https://inkscape.org) for was used for graph rendering.

### Prolactin

At baseline, to compare the association of prolactin levels with PANSS and MADRS between the two age groups, we performed separate Spearman’s rho correlations between continuous prolactin levels and symptoms scores in women below and above 40. In the longitudinal analysis, we performed separate Spearman’s rho correlations between over-time changes in prolactin levels and changes in symptoms scores in women below and above 40. We also explored the differences in prolactin levels in the four age-by-diagnosis groups, both at baseline and in longitudinal analyses.

Corrections for multiple comparisons across the different clinical scales used were not employed, not only because this study was a secondary analysis, but also because PANSS, PANSS subscales and MADRS are all strongly correlated with each other. Results in the cross-sectional analysis showed consistency between PANSS and its subscales, suggesting that findings were unlikely to be due to mere chance.^28^

## Results

### Descriptive data

After excluding two women older than 40 prescribed with HRT, the sample included *N* = 174 participants, with mean age ± s.d. = 42.8 ± 8.8 years. All were of female sex, although they were not asked about gender identity. Data on ethnicity were available for 170: *N* = 8 Asian, *N* = 57 Black, *N* = 1 Chinese, *N* = 5 Mixed, *N* = 92 White and *N* = 7 Other. In terms of clinical diagnosis (ICD-10), 99 women had schizophrenia, 2 had delusional disorder, 2 had acute psychotic disorder, 39 had schizoaffective disorder, 4 had unspecified non-organic psychotic disorder, 17 had bipolar disorder with psychotic symptoms and 10 had major depressive disorder with psychotic symptoms. The mean duration of antipsychotic treatment in the whole sample was 174.3 ± 27.6 days, with a range of 30 to 182 days.

Women aged below (*N* = 65) and above (*N* = 109) 40 did not significantly differ in baseline prolactin levels, BMI, C-reactive protein, smoking habits, rates of hyperprolactinaemia, proportion of affective versus non-affective diagnosis, proportion of prolactin-raising antipsychotics, dose equivalents of antipsychotics and proportion of antipsychotics metabolised by CYP 1A2 (as opposed for 3A4 – for those on antipsychotic monotherapy only) (Table 1). There were no differences in drug use between the two age groups (including opiates, cocaine, amphetamine, methamphetamine and cannabis). As expected, the two age groups differed for duration of illness (*p* < 0.001, see Table 1).

### Cross-sectional analysis

#### Relationship between age and clinical symptoms

**Fig. 1:**
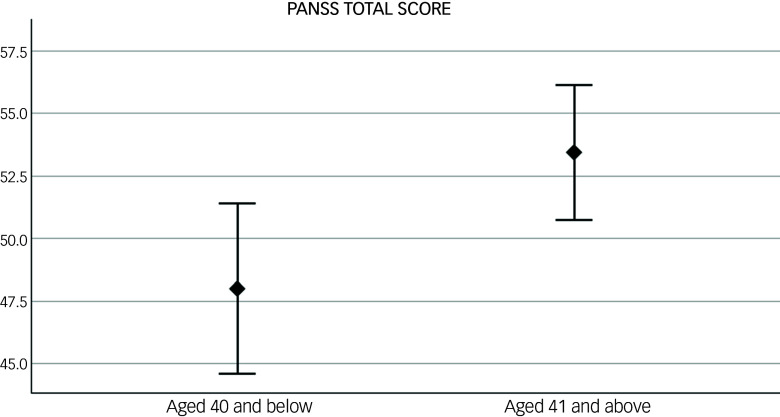
Difference in baseline psychotic symptoms, measured with the Positive and Negative Syndrome Scale (PANSS) total score, between women under (*N* = 63) and over 40 (*N* = 100). *p* = 0.01. Error bar: 95% CI.

At baseline, women aged >40 had higher PANSS total scores compared with those aged ≤40. When looking at PANSS symptom domains, similar results were found for PANSS general symptoms with just trend levels of significance for positive and negative symptoms (see Table 2 and Fig. 1).

**Table 2:** Baseline descriptive statistics for clinical symptoms according to age group

Symptom	Age ≤ 40		Age > 40		Statistics (Mann–Whitney)
	*N*	Mean ± s.d.	*N*	Mean ± s.d.	
PANSS total	63	48.0 ± 13.03	100	53.45 ± 14.14	***p* = 0.014** ***U* = 2433.0** ***z* = −2.4**
PANSS positive	63	10.65 ± 3.56	103	12.32 ± 5.27	*p* = 0.06 *U* = 2666.5
PANSS negative	63	11.63 ± 4.14	100	13.22 ± 5.42	*p* = 0.08 *U* = 2643.5
PANSS general	63	25.71 ± 7.79	103	28.04 ± 7.38	***p* = 0.03** ***U* = 2591.0** ***z* = −2.2**
MADRS total	64	10.78 ± 9.67	103	12.30 ± 9.03	*p* > 0.05

PANSS, Positive and Negative Syndrome Scale; MADRS, Montgomery Asberg Depression Rating Scale.

Significant statistics are in bold.

The duration of illness only showed a trend of correlation with baseline PANSS total scores (*R* ^2^ = 0.02, *p* = 0.076). In the backward stepwise linear regression with PANSS as dependent variable and age (dichotomic, below and above 40) and duration of illness (DOI) as predictor, the first model (adjusted *R* ^2^ = 0.09, *F* = 2.8 and *p* = 0.07), included both DOI (*B* = 0.004, *p* = 0.97 and age (*B* = 11.4, *p* = 0.03) as predictors. In the following step, where DOI was excluded from the model, only age was left as a predictor (adjusted *R* ^2^ = 0.12, *F* = 5.8 and *p* = 0.02), suggesting that only age had a significant predictive effect on PANSS scores.

**Fig. 2:**
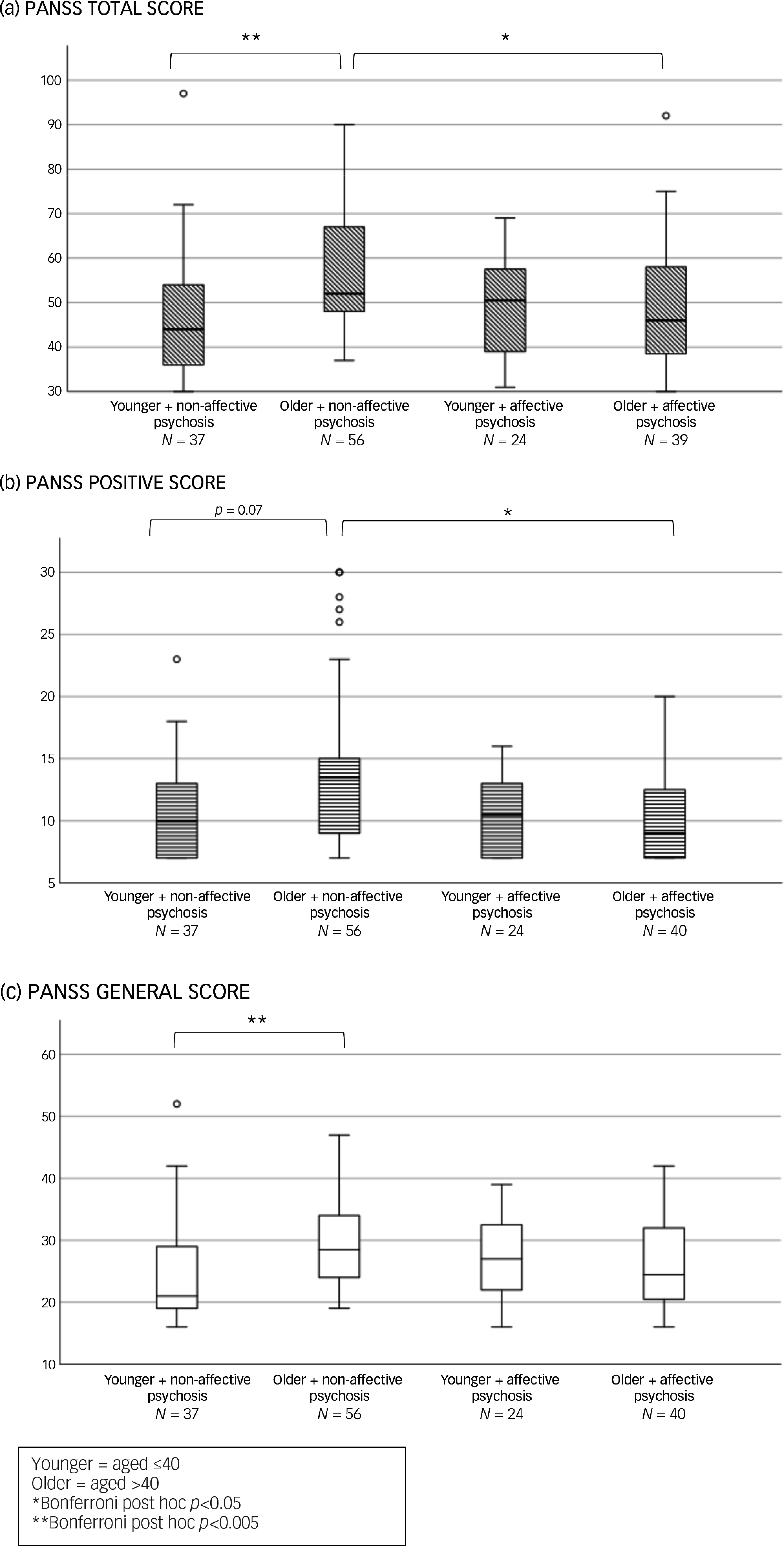
Baseline differences according to age and diagnosis in Positive and Negative Syndrome Scale (PANSS) total (a), positive (b) and general score (c). Bar chart shows median and interquartile ranges.

Our sub-analysis comparing affective to non-affective psychoses (see Fig. 2) showed an overall significant difference among the four age X diagnosis groups in PANSS total scores (H(d.f. = 3) = 16.8, *p* < 0.001) and in the positive (H(3) = 11.8, *p* = 0.008) and general symptoms domains (H(3) = 12.6, *p* = 0.006). Post hoc analyses revealed that women aged >40 with non-affective psychosis had significantly higher PANSS total and positive scores compared with their age peers with affective psychosis (*p* < 0.05). Older women with non-affective psychosis also had higher PANSS positive (trend levels of significance *p* = 0.072), total and general scores than their younger counterparts with non-affective psychosis (*p* < 0.005) (Fig. 2).

#### Relationship to antipsychotic metabolism pathways

**Fig. 3:**
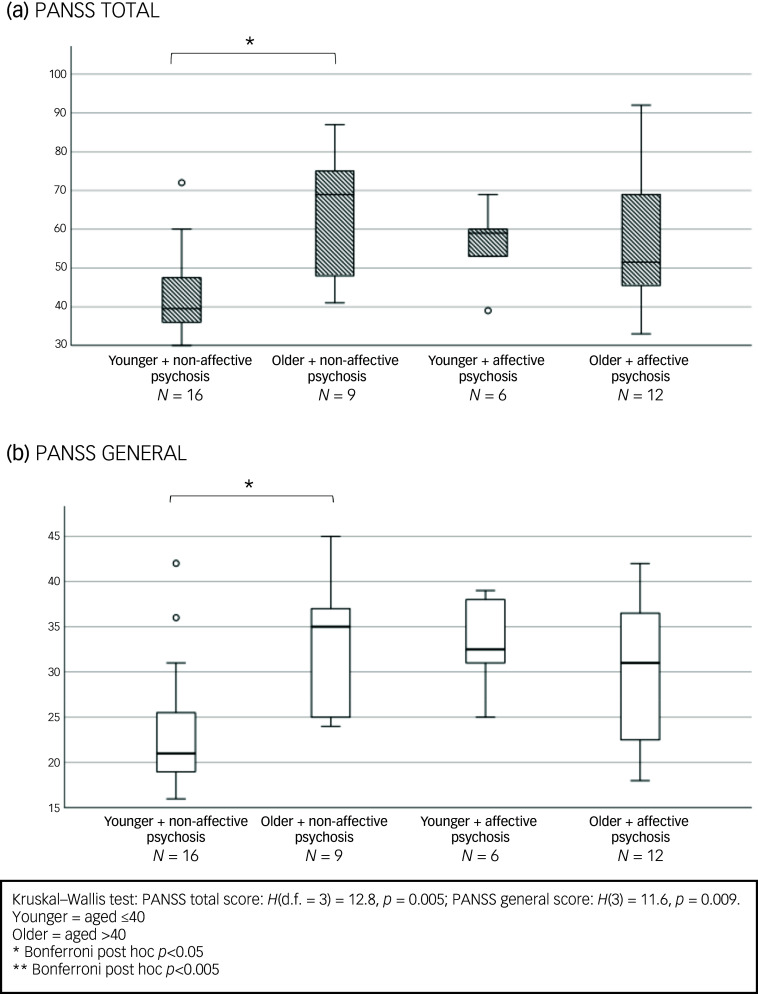
Baseline clinical symptoms differences according to age and diagnosis in women treated with olanzapine or clozapine.

In the subgroup treated with clozapine or olanzapine (metabolised by CYP 1A2), older women with non-affective psychosis had higher PANSS total and general scores than their younger, non-affective counterparts (see Fig. 3(a) and (b)). Not surprisingly, among younger women, those with affective psychosis had higher MADRS scores than their age peers with non-affective psychoses (see Fig. S1 in the Supplementary Material available at https://doi.org/10.1192/bjo.2025.10860).

We found no difference in clinical symptoms according to age and/or diagnosis in women on monotherapy with antipsychotics partially or totally metabolised by CYP 3A4.

#### Effect of prolactin levels

We found no significant correlation between continuous values of prolactin and baseline clinical symptoms scores across the whole sample or in the age subgroups, in the diagnostic subgroups and in the age by diagnosis subgroups. However, among women with moderate hyperprolactinaemia (levels above 1000 mlU/L, *N* = 9), those aged above 40 had higher PANSS positive scores than their younger counterparts (*N* = 12) (Mann–Whitney *U* = 26.5, *p* = 0.049 – mean ± s.d.: 15.1 ± 6.2 *v.* 10.2 ± 2.7), with comparable doses of prolactin-raising antipsychotics between the two age groups.

### Longitudinal analyses

We found no significant difference in change over time of PANSS total scores between the two age groups. However, women above 40 showed slightly decreasing negative symptoms scores over time (Wilcoxon *z* = −2.1, *p* = 0.03; descriptive data in Table 3 in the Supplementary Material).

Changes in clinical scores (for all PANSS domains and MADRS) in both age groups were not associated with changes in prolactin levels over time, nor with the metabolism pathway of antipsychotic medication (1A2 versus 3A4) nor being in a specific trial arm for the IMPaCT RCT (treatment as usual versus IMPaCT therapy). We found no significant effect of diagnosis (affective versus non-affective) and diagnosis-by-age on our longitudinal analysis.

All the above cross-sectional and longitudinal presented results were confirmed by parametric tests.

## Discussion

In this work, we provide descriptive and statistical data on the association between age and clinical symptoms in women with psychosis and investigate the relationship with different antipsychotics and hyperprolactinaemia.

Women aged over 40 had higher baseline PANSS total scores than their younger counterparts. This was also significant in the PANSS general subscale, which measures somatic concern, anxiety, guilt feelings, tension, mannerisms and posturing, depression, motor impairment, uncooperativeness, unusual thought content, disorientation, poor attention, lack of judgment and insight, disturbance of volition, poor impulse control, preoccupation and active social avoidance. Interestingly, this age-related difference was not observed in women with affective psychosis, suggesting a potential specificity to non-affective diagnostic subtypes. The difference was more pronounced in the subset of women on monotherapy with olanzapine and clozapine, both metabolised through CYP 1A2, with older women with non-affective psychosis showing higher PANSS scores than their younger counterparts across the total and general domains.

This change in clinical presentation may be at least partially related to the biological changes occurring with menopause. Menopause usually starts in the mid-forties, with an average menopause age of 50,^14^ but perimenopause starts a number of years earlier.^29^ During the perimenopausal years, in addition to hormone deficiency, fluctuation in hormone levels can be a significant factor affecting clinical presentation.^30^

In schizophrenia, the transition to menopause has been associated with exacerbation of psychotic, mood and anxiety symptoms,^5^ consistent with our findings, which could be reflective of perimenopause and menopause. Moreover, the reduction in circulating levels of oestrogen that characterises this life stage exerts negative effects on physical health (including osteoporosis and cardiovascular risk). It may also be a time of social change. Resulting changes in quality of life^15^ could contribute to the worse PANSS general symptoms seen here and, overall, a poorer response to treatment.^2^

Our results are in line with those of Sommer et al, who found higher hospitalisation rates in women in Finland with schizophrenia-spectrum disorders aged above 45 compared with their younger counterparts and with their male peers.^4^ The authors found similar results when focusing separately on patients in monotherapy with clozapine or olanzapine, highlighting how, even when doses of these medications were high, older women were still more vulnerable to relapse. The age threshold of 40 years in our study is designed to reflect the more diverse ethnicity of our sample, which included 40% of Black women and 5% of Asian women, for whom menopause transition can start earlier than in White women.^16,17,31^ Nevertheless, in line with Sommer’s results, in our sample women aged above 45 had more severe clinical symptoms (higher total PANSS scores) than those below 40 (*U* = 1734.5 *p* = 0.04).

The specificity of our results for women with non-affective psychosis may align with the poorer long-term outcomes of non-affective psychoses like schizophrenia compared with schizoaffective disorder and psychotic affective disorders.^32^

Our findings could also suggest differential neuroprotective effects of oestrogen in women with non-affective than affective psychoses. Oestrogen has modulating effects on dopaminergic activity, particularly in the mesolimbic circuits implicated in schizophrenia. Post-menopausal oestrogen decline may therefore unmask or exacerbate underlying dopaminergic dysregulation, with deterioration in positive and general symptoms.^33^ In contrast, affective psychoses are more strongly influenced by serotonergic and mood-related pathways, where oestrogen plays a less direct role in modulation.^34^

We found no difference in prolactin levels between age groups and diagnostic groups, either cross-sectionally and longitudinally. We found no correlation between prolactin and clinical symptoms, although we did not measure cognitive functioning like Jordá-Baleri et al, who found a correlation between working memory performance and prolactin levels in FEP women.^8^

However, in our sample, among women with hyperprolactinaemia, older women had higher baseline positive symptom scores than their younger counterparts despite comparable doses of antipsychotic medications. By contrast, longitudinal clinical outcomes were not worse. A possible explanation is that in women above 40, the hypoestrogenic state due to iatrogenic hyperprolactinaemia is further exacerbated by age-related reductions in oestrogen, reflected in worse clinical symptoms. However, further research is needed to explore and confirm this hypothesis. Increasing attention is being directed at the interaction between hyperprolactinaemia and oestrogen levels and how this has an impact on symptom severity in schizophrenia,^35^ but the additional role of age and menopause in this context is still understudied. There is no evidence that our findings were affected by the clinical trial intervention.

### Clinical implications

Overall, our study highlights the importance of taking life stage and the hypothalamus-pituitary-gonadal axis into account when assessing women’s mental health. Screening for perimenopausal/menopausal symptoms should be part of routine care, given their important effects on both mental and physical health.^36^ Indeed, unidentified and untreated menopause is associated with worsened mental health, which in turn may lead to reduced access to or uptake of physical health care.^3^ It is notable that only two women (<2%) in the ‘older group’ were excluded from analysis because they were on HRT, highlighting how few women accessed hormonal treatment for menopausal symptoms.

Of note, prolonged hyperprolactinaemia of over 5 years has been associated with an increased risk of breast cancer in women with schizophrenia.^37,38^ Overall, when possible, prolactin-sparing medications should be preferred when treating women with psychosis of all ages. Moreover, older women may need further adjustments because of menopausal hormonal changes, cognisant of CYP 450 metabolism pathways.^26^

Importantly, lowered oestrogen levels post-menopause can reduce the sensitivity of D2 receptors to antipsychotics. Therefore, increasing the dose of these medications may not necessarily improve clinical outcomes.^4^ The National Institute of Health and Care Excellence and others recommend an individualised approach to menopause management, including advice on exercise, weight, smoking cessation and reducing alcohol consumption, together with HRT when indicated.^39^ Trials of antipsychotic augmentation with oestradiol^5^ or with the selective oestrogen receptor modulator raloxifene in women with psychosis were superior to placebo in treating positive, negative and general symptoms of schizophrenia,^40,41^ especially in postmenopausal women.^42–44^ Brand and colleagues recently showed in a Finnish cohort of women that menopausal hormone therapy was associated with a lower relapse risk for psychosis as compared to non-use and was specifically effective in preventing relapse when started before the age of 56.^45^ Further studies are needed to explore whether replacing hormone deficiency directly during the menopause transition overall benefits women’s mental and physical health and if hormonal therapy can counteract the wider negative effects of hyperprolactinaemia.

The main strengths of our study are its relatively large sample size and the possibility to correct for several factors including BMI, inflammation (C-reactive protein) and habits and the longitudinal design. This allowed us to follow-up clinical and biological changes over time.

This study also had some limitations. Missing data limited the analysis of multiple factors affecting the same dependent variable. For example, it was not possible to test for an interaction between age and antipsychotic type or prolactin levels, and so conclusions about the differential effects of hyperprolactinaemia are based solely on differences between age groups. Moreover, we did not employ a dedicated questionnaire to identify menopause but used information on age as a proxy for it.

Future longitudinal studies should use specific clinical measures to capture menopause symptoms and psychiatric symptoms (including treatment response) at this critical juncture. Overall, despite such limitations, we were able to replicate existing results, to produce relevant findings to guide future research in women’s mental health and to highlight gaps to be filled in everyday care, as well as provide a numerical basis for future clinical research in this important area.

In conclusion, women with non-affective psychosis aged above 40 may be more susceptible to clinical deterioration, at least partially due to the menopausal transition and the additional hormonal impact of iatrogenic hyperprolactinaemia. Identifying and managing hormonal changes, considering ethnic diversity, and adjusting psychotropic medications accordingly, seems vital for a more personalised treatment approach for women’s mental and physical health.

## Supporting information

Nettis et al. supplementary material 1 Nettis et al. supplementary material

Nettis et al. supplementary material 2 Nettis et al. supplementary material

## Data Availability

The dataset used and/or analysed during the current study is available from the corresponding author on reasonable request.

## Supplementary material

The supplementary material is available online at https://doi.org/10.1192/bjo.2025.10860
