# Supplementary material for: The effect of age on the clinical presentation and treatment of women with psychosis: secondary analysis of the IMPaCT Randomised Clinical Trial
Source: BJPsych Open. 2025 Dec 2;12(1):e5. doi: 10.1192/bjo.2025.10860 (PMC12724110; doi:10.1192/bjo.2025.10860)
Supplement: Nettis et al. supplementary material 1 — Nettis et al. supplementary material [file S2056472425108600sup001.docx]

**FIG S1-SUPPLEMENTAL material: baseline MADRS score differences according to age and diagnosis in women treated with olanzapine or clozapine.**

BASELINE MADRS SCORE


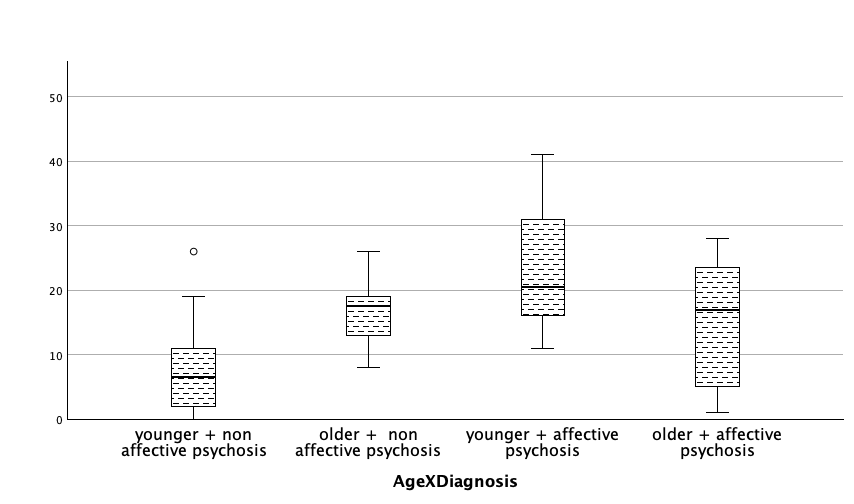


*

N=16 N=10 N=6 N=12

Kruskar Wallis test: MADRS score (H(3) = 11.4, p=0.009).

younger = aged <=40

older =aged >40

* Bonferroni post-hoc p<0.05
