# Supplementary material for: The effect of age on the clinical presentation and treatment of women with psychosis: secondary analysis of the IMPaCT Randomised Clinical Trial
Source: BJPsych Open. 2025 Dec 2;12(1):e5. doi: 10.1192/bjo.2025.10860 (PMC12724110; doi:10.1192/bjo.2025.10860)
Supplement: Nettis et al. supplementary material 2 — Nettis et al. supplementary material [file S2056472425108600sup002.docx]

***Supplemental Materials***

***Table 3 Descriptive longitudinal data on clinical and physical variables. The Between groups statistics of the change (deltas) of symptoms over time (Mann-Whitney) are presented on the far-right column. Significant (p<0.05) within-group changes from baseline to follow-up (Wilcoxon) are in bold font.***

|  |  | **Age <40** | **N of pairs BL/FU for within-group tests** |  | **Age >40** | **N of pairs BL/FU for within-group tests** | **Between groups stats: difference between Deltas** |
| --- | --- | --- | --- | --- | --- | --- | --- |
| PANSS TOT  (mean±SD) | BL | 49.5± 13.8 | N=41 | BL | 53.7 ±14.5 | N=83 | p>0.05 |
|  | FU | 50.6±15.4 |  | FU | 50.9±13.6 |  |  |
| PANSS POS  (mean±SD) | BL | **10.9 ± 3.6** | N=41 | BL | 12.5 ±5.4 | N=85 | U=1319.5  p=0.03* |
|  | FU | **11.9±4.1** |  | FU | 12.4±5.4 |  |  |
| PANSS NEG  (mean±SD) | BL | 11.5 ±4.1 | N=42 | BL | **13.1 ±5.2** | N=83 | U=1390.5  p=0.06 |
|  | FU | 12.3±5.6 |  | FU | **12.1±4.7** |  |  |
| PANSS GEN  (mean±SD) | BL | 26.9 ±8.2 | N=42 | BL | 28.2 ±7.7 | N=86 | P>0.05 |
|  | FU | 26.3±8.4 |  | FU | 26.9±7.2 |  |  |
| MADRS  (mean±SD) | BL | 11.7 ±10.4 | N=42 | BL | 12.6 ±9.2 | N=88 | P>0.05 |
|  | FU | 13.1±11.1 |  | FU | 12.9±9.9 |  |  |
| PRL  (mean±SD) | BL | 602.5±940.1 | N=27 | BL | 457.2±510.8 | N=52 | P>0.05 |
|  | FU | 533.2±522.4 |  | FU | 632.2±877.1 |  |  |
| BMI  (mean±SD) | BL | 32.00±7.4 | N=41 | BL | 34.6±9.6 | N=78 | P>0.05 |
|  | FU | 32.8±6.9 |  | FU | 33.8±9.5 |  |  |
| Olanzapine equivalents  (mean±SD) | BL | 18.4±9.1 | N=36 | BL | 18.4±13.6 | N=73 | P>0.05 |
|  | FU | 18.2±9.2 |  | FU | 19.2±13.0 |  |  |

BL=baseline

FU= combined follow-up at 15-18 months

PANSS TOT=PANSS total score

PANSS POS= PANSS positive symptoms score

PANSS NEG= PANSS negative symptoms score

PANSS GEN= PANSS general symptoms score

PRL=prolactin levels

*not confirmed by parametric tests
